# Multiple concurrent opportunistic infections in patient with myasthenia gravis: A case report

**DOI:** 10.1080/21505594.2025.2545570

**Published:** 2025-08-28

**Authors:** Jingrou Chen, Jingchun Fang, Li Sun, Zongjun Zhang, Qinghua Ma, Jiahao Wu, Yili Chen, Kang Liao, Tiandi Long, Hongxu Xu

**Affiliations:** a Department of Laboratory Medicine, the First Affiliated Hospital, Sun Yat-sen University, Guangzhou, China; b Department of Laboratory Medicine, Nansha Division, the First Affiliated Hospital, Sun Yat-sen University, Guangzhou, China; c Department of Pathology, Nansha Division, the First Affiliated Hospital, Sun Yat-sen University, Guangzhou, China; d Department of Laboratory Medicine, Guangdong Province Prevention and Treatment Center for Occupational Diseases, Guangzhou, China; e Department of Laboratory Medicine, Zhujiang Hospital of Southern Medical University, Guangzhou, China

**Keywords:** Myasthenia gravis, multiple opportunistic infections, complex evaluation, case report

## Abstract

Myasthenia gravis (MG), a rare autoimmune disorder with poor prognosis, especially when complicated by opportunistic infections, which pose significant risks in clinical practice. We aimed to analyse a clinical case of a middle-aged male patient with MG, who developed severe lower gastrointestinal bleeding and multiple opportunistic infections post-immunosuppressive therapy. This case report is based on comprehensive clinical evaluations, including colonoscopy, histopathological examination, bronchoscopy, bronchoalveolar lavage (BAL), and metagenomic next-generation sequencing (mNGS). The patient exhibited persistent ptosis and pulmonary infection, and was treated with Meropenem 12 mg once daily (qd), Tacrolimus 2 mg qd, and Bromhexine 60 mg three times daily (tid). Due to ongoing lower gastrointestinal bleeding, surgery was performed. Colonoscopy revealed multiple ulcers, with histopathology confirming Cytomegalovirus (CMV) and Histoplasmosis infections. Bronchoalveolar lavage fluid (BALF) identified infections with Aspergillus fumigatus, Talaromyces, and Stenotrophomonas maltophilia. mNGS further detected Pneumocystis jirovecii. Based on these findings, the treatment plan was adjusted to include Amphotericin B complex 25 mg via intravenous (IV) qd, Tigecycline 100 mg q12h, and Sulfamethoxazole (SMZ) 0.96 g q6h for anti-infection, along with Ganciclovir 250 mg IV q12h. The patient continues to receive infusions of immunoglobulins and albumin. This case underscores the importance of monitoring MG patients on immunosuppressive therapy for opportunistic infections, emphasizing the complexity of managing multiple pathogens simultaneously.

## Introduction

MG is an autoimmune disease that affects the neuromuscular junction, characterized by muscle fatigue and weakness. Patients often require long-term immunosuppressive therapy to control symptoms [1]. However, immunosuppressive therapy inhibits pathological autoimmune responses and weakens the body’s defence against various pathogens, increasing the risk of opportunistic infections [2]. Opportunistic infections are relatively common in immunosuppressed patients, such as cryptococcosis [3], dermatophytosis [4], Pneumocystis jirovecii pneumonia (PJP) [5], and CMV infection [6], but simultaneous infections with multiple pathogens are sporadic.

CMV is widely prevalent in humans and other mammals. It belongs to the Herpesviridae family and is a large DNA virus. In immunocompetent individuals, CMV infection is usually asymptomatic, but in immunocompromised individuals, especially those with severely impaired immune systems, CMV infection can lead to serious complications including loss of graft and death [7].

Histoplasma is a dimorphic fungus that exists as spores in the environment and transforms into a yeast form within the body. Its characteristic yeast cells are small (2–4 micrometres) and possess a polysaccharide capsule, one of its virulence factors [8]. The capsule helps the fungus survive and replicate within host cells while inhibiting phagocytosis by immune cells [9]. This fungus is widely distributed in many regions worldwide, especially in certain areas of North America, such as the Mississippi and Ohio River valleys, known as the “histoplasmosis belt” [10]. This fungus was encountered for the first time at our hospital since its opening. When inhaled by humans, these spores can cause infection. In healthy individuals, initial infection is usually asymptomatic or presents as a mild, flu-like illness. However, in immunosuppressed patients, Histoplasma capsulatum can rapidly disseminate throughout the body, causing severe systemic infection known as disseminated histoplasmosis [11]. Disseminated histoplasmosis can affect multiple organs, including the lungs, liver, spleen, heart, kidneys, bone marrow, and central nervous system, potentially leading to severe complications such as pneumonia, liver failure, splenomegaly with hyperfunction, endocarditis, nephritis, bone marrow suppression, and meningitis, which can be life-threatening if not treated promptly [12].

Aspergillus fumigatus is a common fungus widely present in the natural environment, such as soil, plants, and airborne particles. Pulmonary aspergillosis is a serious opportunistic infection that can occur when immunocompromised individuals inhale Aspergillus spores. Normally, inhalation of small amounts of Aspergillus spores does not cause disease, but in recent years, there has been an increase in infections among individuals with mild immunosuppression [13,14].

T. marneffei, formerly known as Penicillium marneffei, is a thermally dimorphic fungus endemic to Southeast Asia, particularly in southern China and northern Vietnam. It primarily enters the body through the respiratory tract, leading to talaromycosis, a systemic infection mainly affecting immunosuppressed patients [15].

Stenotrophomonas maltophilia is a common Gram-negative bacillus widely recognized as a major nosocomial pathogen. It is usually found in soil, water, plants, and animals and can survive for extended periods in moist environments [16]. It primarily affects immunocompromised patients, causing infections such as pneumonia [17], sepsis [18], meningitis [19], and urinary tract infections [20].

Pneumocystis jirovecii, formerly known as Pneumocystis carinii, is a unique microorganism that primarily causes lung infections in immunosuppressed patients, known as Pneumocystis pneumonia (PCP). Although PCP is more common in HIV/AIDS patients, it can also occur in other immunosuppressed populations [21,22].

The patient in this case received long-term immunosuppressive therapy for MG, which likely contributed to decreased immune defence mechanisms and increased susceptibility to opportunistic infections [23]. The patient was concurrently infected with CMV, Histoplasma, Aspergillus fumigatus, T. marneffei, Stenotrophomonas maltophilia, and Pneumocystis jirovecii, a situation rarely reported in the medical literature, further highlighting the complexity and severity of multiple fungal infections in immunosuppressed patients [24]. However, interpreting mNGS results posed a significant diagnostic challenge, particularly when discrepancies arose between sequencing data and clinical evidence. In this case, for example, high Epstein – Barr virus (EBV) sequence reads were detected without serological confirmation of active infection, while low Candida albicans reads were identified but could not be verified through gold-standard culture.

## Case description

### Case presentation

This study enrolled one patient from the inpatient neurology unit of Nansha Division, the First Affiliated Hospital of Sun Yat-sen University. Selection criteria included individuals with a confirmed diagnosis of MG based on established diagnostic guidelines, who had received immunosuppressive therapy and developed severe infections necessitating hospitalization. Comprehensive clinical evaluations were conducted upon admission to ensure robust data collection.

A 45-year-old male patient was admitted urgently with a chief complaint of “drooping eyelid for 11 months and stomach bleeding for 6 days.” Diagnosed with MG in February 2023, he began immunosuppressive therapy with methylprednisolone 12 mg qd, tacrolimus 2 mg qd, and pyridostigmine 60 mg tid, but symptom control remained poor. Symptoms included intermittent eyelid drooping, blurred vision, slurred speech, limb fatigue, muscle soreness, difficulty standing, and occasional swallowing issues. In April 2023, the patient contracted H1N1 influenza, presenting with fever, respiratory distress, and limb weakness, and was diagnosed with a “myasthenic crisis.” Three months prior, the patient experienced recurrent pulmonary infections. On 6 November 2023, a pulmonary artery CTA revealed multiple small patchy low-density filling defects in the main pulmonary arteries of both lungs, the apical posterior segment of the right upper lobe, the middle lobe of the right lung, and the pulmonary artery branches of both lower lobes, indicating multiple thrombi. Heparin anticoagulation therapy was initiated; however, gastrointestinal bleeding occurred during the treatment. On 21 November 2023, BALF mNGS results from another hospital identified the presence of Stenotrophomonas maltophilia, Enterococcus faecalis, Pneumocystis jirovecii, and T. marneffei. Before this admission, he presented to the emergency department due to worsening gastrointestinal bleeding, accompanied by abdominal pain, bloating, diarrhoea, loose stools, and chest pain. Given his complex history, comprehensive evaluation and urgent management were required. The study was approved by The First Affiliated Hospital of Sun Yat sen University and was in line with the Declaration of Helsinki. This study adhered to the CARE guidelines.

### Physical examination

**Figure 1.**
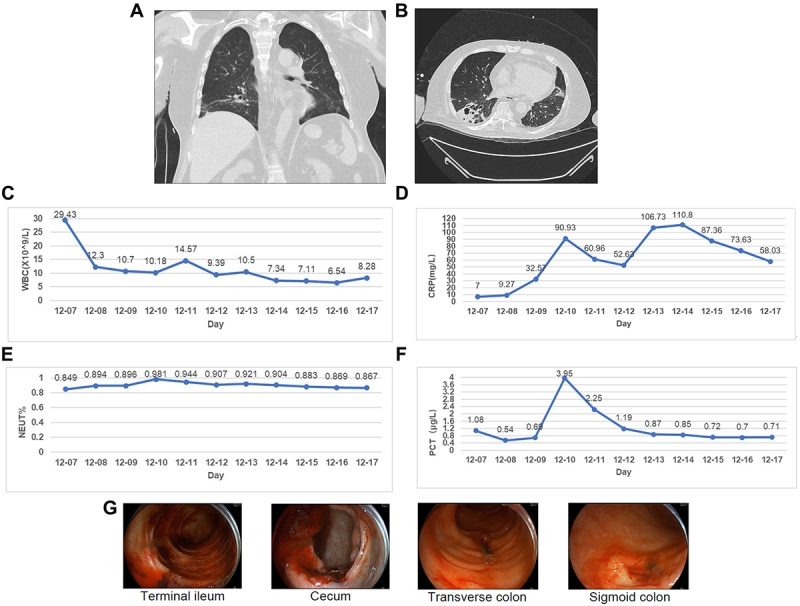
Results of auxiliary examinations. (A) the image shows multiple patchy areas of increased density in both lungs, with bronchiectasis in the lower lobes and subsegmental bronchi. The internal diameter is larger than the accompanying bronchial artery branches, presenting as cylindrical, saccular, and mixed types, more pronounced in the right lung. (B) the image shows multiple parenchymal nodules in both lungs. (C–F) clinical test results. (C) trend of WBC count (normal range for WBC: 4.0-10x10^9/L). (D)trend of NEUT % (normal range for NEUT%: 0.460–0.750). (E) trend of CRP (normal range for CRP: 0–10 mg/L). (F) trend of PCT (normal range for PCT: 0.00–0.05 μg/L). (G) colonoscopy examination.

Upon admission, physical examination revealed the following: body temperature 36.5°C, pulse rate 120 beats per minute, respiratory rate 20 breaths per minute, and blood pressure 90/60 mmHg. The patient appeared anaemic. Cardiac and pulmonary auscultation showed no abnormalities; however, coarse breath sounds and both dry and wet crackles were heard in both lungs, indicating a lung infection. Based on the results of a chest CT scan, revealed bronchiectasis in the lower lobes (more prominent on the right) with thickened bronchial walls, peripheral high-density shadows, multiple solid nodules with clear margins, occasional spiculation, pleural traction, and scattered patchy opacities (Figure 1A,B), the patient was diagnosed with pneumonia. Abdominal examination showed no significant tenderness, rebound tenderness, or palpable masses, and bowel sounds were normal.

### Auxiliary examinations

Complete blood count revealed elevated white blood cells (WBC)29x10^9/L and neutrophils (NEUT) percentage 84.9%, increased C-reactive protein (CRP) 7 mg/L, and elevated procalcitonin (PCT) 1.08 μg/L (Figure 1C–F), suggesting a possible infection or inflammation. Coagulation tests indicated prolonged prothrombin time (Supplementary Table 1), consistent with gastrointestinal bleeding. The patient was tested for syphilis, hepatitis B antibodies, HIV, respiratory pathogens (including SARS-CoV-2, influenza viruses, RSV, adenoviruses, human rhinoviruses, and Mycoplasma pneumoniae), and Mycobacterium tuberculosis by PCR, all of which were negative. Cellular immunity chip testing showed decreased levels of CD3, CD4, and CD8, indicating severe cellular immunodeficiency (Supplementary Table 2).

### Endoscopic examination

Gastroscopy and colonoscopy revealed abundant dark red and fresh blood, along with residual blood clots. There was no active bleeding at the terminal ileum. Large deep ulcers with partial oozing blood were observed at the caecum, approximately 40 cm and 15 cm from the anal verge, with a suspected fistula observed at one site. The haemostatic powder was applied to the ulcerated areas to control bleeding. These findings suggest multiple ulcerative colitis with bleeding (Figure 1G).

### Pathological examination

**Figure 2.**
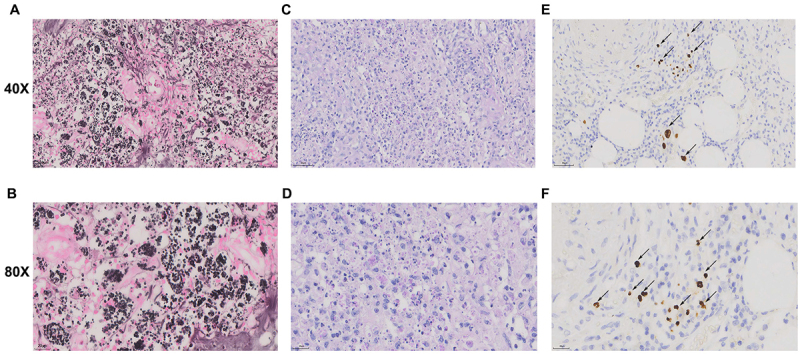
Intestinal CMV virus and tissue cytoplasmic bacterial infection. (A, B) gomori methenamine silver stain demonstrating clustered spherical to oval Histoplasma organisms within multinucleated giant cells, with most organisms appearing as darkly stained dots. (C, D) PAS stain showing clear outlines of spores with red-stained capsules and non-staining cell contents. (E, F) cells affected by CMV with uniformly deep brown nuclear staining, sometimes exhibiting diffuse nuclear and cytoplasmic staining.

Histopathological examination revealed transmural inflammation of the intestinal wall with cellular aggregation and granuloma formation. Staining with Gomori methenamine silver (GMS) and periodic acid-Schiff (PAS) suggested Histoplasma infection (Figure 2A-D). Immunohistochemistry showed the presence of inclusion bodies (Figure 2E,F), and quantitative CMV DNA in venous blood was 2.56x10^3, indicating CMV infection.

### Molecular microbiology examination

**Figure 3.**
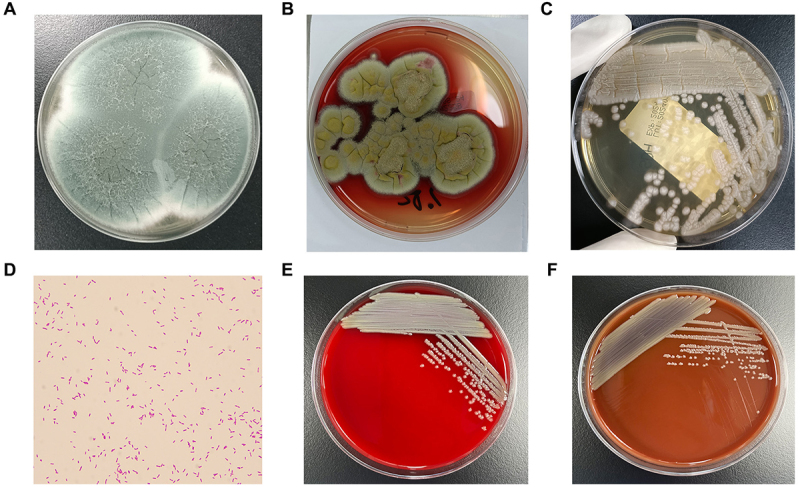
Microorganisms cultured from BALF. (A) Aspergillus fumigatus cultured on Sabouraud agar at 35°C for 5 days, the center of the colonies gradually turns grayish-green or bluish-green with a white edge. (B, C) T. marneffei cultured at 28°C and 35°C for 5 days. B: At 28°C, the colony morphology was velvety, and the wine-red pigment diffused into the culture medium. C: At 35°C, the characteristic yeast-like fungal morphology was observed under a microscope. (D–F) morphology of Stenotrophomonas maltophilia. (D) gram-negative rods, with polar flagella, motile, non-spore-forming. (E, F) on blood agar and chocolate agar, colonies are round, smooth, moist, and light yellow after 18–24 hours of incubation at 35°C.

Fungal glucan test (G test): 504.56 pg/mL. Aspergillus antigen in bronchoalveolar lavage fluid (BALF): 6.900. Microscopic examination: Found fungal hyphae (suspected Aspergillus hyphae). Culture of BALF revealed Aspergillus fumigatus (Figure 3A), Talaromyces marneffei (Figure 3B,C), and Stenotrophomonas maltophilia (Figure 3D-F). Metagenomic next-generation sequencing (mNGS) of BALF identified cytomegalovirus (CMV, sequence count 37,901), Epstein-Barr virus (EBV, sequence count 6516), Aspergillus fumigatus (sequence count 6715), T. marneffei (sequence count 2488), Stenotrophomonas maltophilia (sequence count 1112), and Pneumocystis jirovecii (sequence count 1103) (Supplementary Table 3).

## Treatment course

### Initial treatment and emergency management

Upon admission, the patient received immunosuppressive therapy with pyridostigmine and tacrolimus for myasthenia gravis. Emergency management of gastrointestinal bleeding included blood transfusion, the application of haemostatic powder, the administration of Schnivit lyophilized powder for injection to control bleeding, and anti-infection treatment. Considering the suspected lung infection, a combination of meropenem (12 mg qd) and linezolid (0.6 mg qd) was administered for anti-infection therapy.

### Adjustment of anti-infection treatment regimen

With the return of pathological histology results, a diagnosis of CMV infection and Histoplasma infection was confirmed. BALF NGS suggested multiple bacterial, fungal, and viral infections, prompting an adjustment in the treatment regimen. The patient was administered tigecycline (100 mg q12h), amphotericin B complex (25 mg IV.drip qd), trimethoprim-sulfamethoxazole (0.96 g P.O q6h), and ganciclovir (250 mg IV.drip q12h) to enhance antibacterial, antifungal, and antiviral effects. Subsequently, the patient’s TBIL rose to 145.2 μmol/L and DBIL to 104.0 μmol/L, indicating elevated bilirubin levels and possible antibiotic-related liver damage. The dosage of trimethoprim-sulfamethoxazole was reduced, and the anti-infection regimen was adjusted to vancomycin (0.5 g IV.drip q12h, initial dose 1.0 g IV.drip qd), piperacillin-tazobactam (4.5 g IV.drip q8h), amphotericin B (150 mg IV.drip qd), trimethoprim-sulfamethoxazole (0.48 g P.O q8h), and ganciclovir (250 mg IV.drip q12h).

### Adjustment of immunosuppressive therapy

Due to severe infectious complications, the immunosuppressive therapy regimen was adjusted. Tacrolimus was suspended, and the corticosteroid dosage was reduced to dexamethasone 5 mg qd to lower the risk of infection exacerbation.

### Nutritional and supportive therapy

The patient was at nutritional risk due to gastrointestinal bleeding and surgical trauma. The treatment regimen included parenteral nutritional support and intravenous immunoglobulin (IVIG) therapy at 0.4 g/Kg for 5 days. Additionally, albumin (30 g IV.drip) was administered to enhance the patient’s immune modulation and reduce the incidence of toxic side effects.

### Surgical treatment

Due to multiple bleeding ulcers in the colon and poor endoscopic haemostasis, the patient underwent exploratory laparotomy. Postoperatively, the patient continued to receive anti-infection and supportive treatment while monitoring haemoglobin levels and stool characteristics.

### Postoperative recovery and treatment

Postoperatively, the patient continued to receive mechanical ventilation support, with adjustments made to the ventilation mode to support respiratory function. As the patient’s condition stabilized, the use of sedative and analgesic medications was gradually reduced, and a spontaneous breathing trial (SBT) was attempted.

### Clinical outcome and pathological results analysis

**Figure 4.**
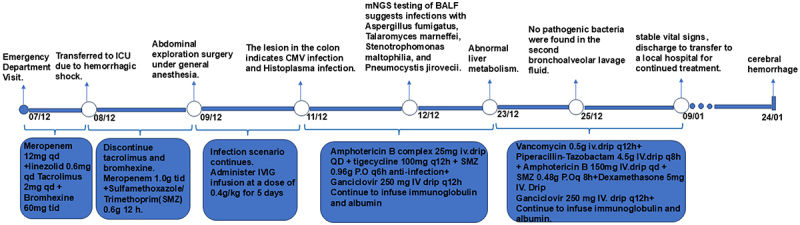
Patient’s treatment timeline.

With comprehensive treatment, the patient was alert, able to follow instructions, afebrile, and breathing smoothly. Gastrointestinal bleeding was controlled, and anaemia improved. On December 25, subsequent BALF testing showed no pathogenic organisms, the patient’s cough lessened, and infection markers gradually declined. The patient continued to receive anti-infection, nutritional support, liver protection, and fasting treatment. However, on 24 January 2024, the patient passed away due to a cerebral haemorrhage. The course of the disease is shown in Figure 4.

## Discussion

Common immunosuppressive agents for MG include tacrolimus, azathioprine, mycophenolate mofetil (MMF), and other therapies [25]. Studies indicate that tacrolimus combined with corticosteroids provides superior clinical outcomes, significantly reducing relapse risk (HR = 0.45, 95% CI 0.25–0.81, *p* = 0.0077) [26,27]. MMF combined with corticosteroids also shows efficacy (HR = 0.32, 95% CI 0.15–0.67, *p* = 0.0020), though its teratogenicity limits use in women of childbearing age [28]. The patient in this case received pyridostigmine and tacrolimus, which effectively suppress the immune system but increase susceptibility to opportunistic infections [29–31]. Emerging therapies like eculizumab and efgartigimod show promise in reducing disability and steroid dependence in generalized MG, potentially benefiting patients with multiple infections [32].

Studies indicate that SARS-CoV-2 increases mortality in MG patients due to respiratory complications [33]. Fortunately, this patient remained uninfected. Upon admission, the patient was diagnosed with multiple opportunistic infections, including CMV, histoplasmosis, Aspergillus fumigatus, Talaromyces marneffei, Stenotrophomonas maltophilia, and Pneumocystis jirovecii. These pathogens are typically not severe in immunocompetent individuals but can cause serious systemic infections in those with compromised immune systems.

CMV is a widespread herpesvirus that is usually asymptomatic in immunocompetent individuals but can cause severe complications such as gastrointestinal ulcers and systemic infections in immunosuppressed patients [34,35]. In immunocompetent individuals, CMV infection is usually asymptomatic or presents with mild symptoms and does not require special treatment. For severe CMV infections in immunosuppressed patients, intravenous ganciclovir is the main treatment. Studies have shown that CMV infection may be associated with the onset and progression of MG. The positive rate of CMV antibodies is relatively high among MG patients, indicating previous CMV infection [36]. Additionally, some studies have found that the presence of CMV DNA in MG patients is associated with the severity of the disease [37]. These findings suggest that CMV infection may play a role in the onset and progression of MG.

The first report of histoplasmosis at the Nansha Hospital of the First Affiliated Hospital of Sun Yat-sen University is significant. Histoplasmosis can quickly disseminate throughout the body in immunosuppressed patients, causing disseminated histoplasmosis [38]. The main treatment for disseminated histoplasmosis is amphotericin B, especially in immunocompromised patients. It is usually administered intravenously for 1–2 weeks. Histoplasmosis is a common opportunistic infection in AIDS patients [39]. In non-AIDS immunosuppressed patients, such as organ transplant recipients or those receiving long-term corticosteroid therapy, histoplasmosis is also an important clinical issue [40]. Histoplasmosis may co-infect with other pathogens, such as CMV [41], as seen in this case. Histoplasmosis in this patient led to severe intestinal ulcers and bleeding.

Aspergillus fumigatus is a common environmental fungus that typically enters the body via the respiratory tract. In immunosuppressed patients, it can cause severe pulmonary and systemic infections [42,43]. Pulmonary aspergillosis is primarily treated with voriconazole, administered intravenously or orally for 6–12 weeks or longer, depending on severity. For patients intolerant or resistant to voriconazole, liposomal amphotericin B is a viable alternative. The diagnostics in this case, similar to early reports [44], relied on Aspergillus fumigatus infections. More recently, molecular techniques have also been utilized for detection. Treatment followed comparable approaches, combining antifungal therapy with adjustments to immunosuppressive medications, emphasizing the necessity of balancing infection control with MG management.

Pneumocystis jirovecii primarily causes Pneumocystis jirovecii pneumonia (PCP) in immunosuppressed patients. Sulfamethoxazole-trimethoprim (SMX-TMP) is the first-line treatment for PCP. Although PCP is more common in HIV/AIDS patients [45], it can also occur in other immunosuppressed populations [46,47]. Jiahui Hou also reported a case of MG complicated with PCP infection [5]. This case describes a 41-year-old male with MG and long-term steroid use, who developed a rare triple co-infection with Pneumocystis jirovecii, Nocardia brasiliensis, and Mycobacterium tuberculosis. Despite advanced diagnostics and treatment, complications including gastrointestinal bleeding, liver impairment, and multiple organ failure led to a poor outcome.

In this case, the patient’s multiple infections complicated both diagnosis and treatment due to overlapping clinical symptoms such as gastrointestinal bleeding and pulmonary infection. A multifaceted diagnostic approach was necessary, involving histopathology, molecular microbiology, and immunohistochemistry to identify and confirm the various pathogens. The treatment plan had to account for the specific characteristics of each pathogen and the patient’s immunosuppressed state. Initial treatment with broad-spectrum anti-infectives was adjusted as pathogen identification became clear. However, certain antibiotics can have side effects in patients with MG. Clarithromycin has been reported to cause transient generalized MG-like symptoms in nonmyasthenic patients, which were responsive to pyridostigmine [48]. Azithromycin was found to be the most common medication associated with MG deterioration in a retrospective study [49]. Fluoroquinolone antibiotics may exacerbate MG in patients with underlying conditions, posing a life-threatening risk [50]. In contrast, cephalosporins, sulpha drugs, clindamycin, tetracyclines, and polymyxin B have not been associated with MG exacerbation and are considered safer options. Therefore, the antibiotics chosen for this patient were among these safer options.

This case highlights the complexity and severity of multiple opportunistic infections in immunosuppressed patients. Multiple infections increase the difficulty of diagnosis and treatment and present higher clinical management demands. To reduce the occurrence of similar cases, monitoring and management of immunosuppressed patients should be strengthened to detect and address infections promptly. Furthermore, this case reminds clinicians to consider the possibility of multiple pathogens in complex infection cases and to adopt comprehensive diagnostic and treatment strategies.

## Supplementary Material

Supplementary Material.docx

## Data Availability

No datasets were generated or analysed during the current study.
