# Supplementary material for: Multiple concurrent opportunistic infections in patient with myasthenia gravis: A case report
Source: Virulence. 2025 Aug 28;16(1):2545570. doi: 10.1080/21505594.2025.2545570 (PMC12396124; doi:10.1080/21505594.2025.2545570)
Supplement: Supplementary Material.docx [file KVIR_A_2545570_SM3901.docx]

**Supplementary Table 1. Coagulation Function Results**

| Item Name | Value | Unit | Normal range | Method |
| --- | --- | --- | --- | --- |
| PT | 15.5 ↑ | s | 11.0-14.0 | Solidification |
| PT% | 73.0 | % | 70.0-130.0 | Solidification |
| INR | 1.21 ↑ |  | 0.80-1.15 | Calculation |
| APTT | 34.7 | s | 28.0-43.0 | Solidification |
| TT | 18.4 | s | 14.0-21.0 | Solidification |
| FIB | 1.72 ↓ | g/L | 2.00-4.00 | Solidification |
| D-D | 1.20 ↑ | mg/L FEU | 0.00-0.50 | Immunoturbidimetry |

Note: APTT, Activated partial thromboplastin time; PT, Prothrombin time; TT, Thrombin time; INR, International standardized ratio of Prothrombin time; FIB, Fibrinogen; D-D, D-Dimer.

**Supplementary Table 2. Cellular immunity chip testing results.**

| Item Name | Value | Unit | Normal range |
| --- | --- | --- | --- |
| Absolute CD4 cell count | 124↓ | pcs/ul | 500-144- |
| Absolute CD8 cell count | 108↓ | pcs/ul | 238-1250 |
| Absolute CD3 cell count | 140↓ | pcs/ul | 770-2860 |
| CD4/CD8 ratio | 1.15 |  | 1.0-2.47 |

**Supplementary Table 3. Pathogens and read counts detected by mNGS in BALF.**

| category | Pathogens | type | Sequence number | Coverage | Relative abundance | Identification Confidence |
| --- | --- | --- | --- | --- | --- | --- |
| bacteria | Stenotrophomonas maltophilia | G- | 1112 | 1.1% | 4.4% | 99% |
| Fungi | Aspergillus fumigatus | - | 6751 | 1.5% | 55.8% | 99% |
|  | Pneumocystis jirovecii | - | 1103 | 0.6% | 24.1% | 99% |
|  | T. marneffei | - | 2488 | 0.4% | 16.4% | 99% |
| Virus | Cytomegalovirus | DNA | 37901 | 99.5% | 80.3% | 99% |
| Parasites | - | - | - | - | - | - |
| Special Pathogens | - | - | - |  | - | - |
| Microecology | Corynebacterium striatum | G+ | 10479 | 17.6% | 75.4% | 99% |
|  | Staphylococcus hominis | G+ | 562 | 1.2% | 5.0% | 99% |
